# Transcriptomic intratumor heterogeneity of breast cancer patient-derived organoids may reflect the unique biological features of the tumor of origin

**DOI:** 10.1186/s13058-023-01617-4

**Published:** 2023-02-21

**Authors:** Sumito Saeki, Kohei Kumegawa, Yoko Takahashi, Liying Yang, Tomo Osako, Mahmut Yasen, Kazutaka Otsuji, Kenichi Miyata, Kaoru Yamakawa, Jun Suzuka, Yuri Sakimoto, Yukinori Ozaki, Toshimi Takano, Takeshi Sano, Tetsuo Noda, Shinji Ohno, Ryoji Yao, Takayuki Ueno, Reo Maruyama

**Affiliations:** 1 Project for Cancer Epigenomics, Cancer Institute, Japanese Foundation for Cancer Research, 3-8-31, Ariake, Koto-ku, Tokyo, 135-8550 Japan; 2 Breast Surgical Oncology, Breast Oncology Center, Cancer Institute Hospital, Japanese Foundation for Cancer Research, Tokyo, Japan; 3 Cancer Cell Diversity Project, NEXT-Ganken Program, Japanese Foundation for Cancer Research, Tokyo, Japan; 4 Division of Pathology, Cancer Institute, Japanese Foundation for Cancer Research, Tokyo, Japan; 5 Cancer Informatics and Biobanking Platform Project, NEXT-Ganken Program, Japanese Foundation for Cancer Research, Tokyo, Japan; 6 Breast Medical Oncology, Breast Oncology Center, Cancer Institute Hospital, Japanese Foundation for Cancer Research, Tokyo, Japan; 7 Department of Gastroenterological Surgery, Gastroenterological Center, Cancer Institute Hospital, Japanese Foundation for Cancer Research, Tokyo, Japan; 8 Director’s Room, Cancer Institute, Japanese Foundation for Cancer Research, Tokyo, Japan; 9 Breast Oncology Center, Cancer Institute Hospital, Japanese Foundation for Cancer Research, Tokyo, Japan; 10 Department of Cell Biology, Cancer Institute, Japanese Foundation for Cancer Research, Tokyo, Japan

**Keywords:** Intratumor heterogeneity, Breast cancer, Patient-derived organoids, scRNA-seq, Cancer cell diversity, Inflammatory breast cancer

## Abstract

**Background:**

The intratumor heterogeneity (ITH) of cancer cells plays an important role in breast cancer resistance and recurrence. To develop better therapeutic strategies, it is necessary to understand the molecular mechanisms underlying ITH and their functional significance. Patient-derived organoids (PDOs) have recently been utilized in cancer research. They can also be used to study ITH as cancer cell diversity is thought to be maintained within the organoid line. However, no reports investigated intratumor transcriptomic heterogeneity in organoids derived from patients with breast cancer. This study aimed to investigate transcriptomic ITH in breast cancer PDOs.

**Methods:**

We established PDO lines from ten patients with breast cancer and performed single-cell transcriptomic analysis. First, we clustered cancer cells for each PDO using the Seurat package. Then, we defined and compared the cluster-specific gene signature (ClustGS) corresponding to each cell cluster in each PDO.

**Results:**

Cancer cells were clustered into 3–6 cell populations with distinct cellular states in each PDO line. We identified 38 clusters with ClustGS in 10 PDO lines and used Jaccard similarity index to compare the similarity of these signatures. We found that 29 signatures could be categorized into 7 shared meta-ClustGSs, such as those related to the cell cycle or epithelial–mesenchymal transition, and 9 signatures were unique to single PDO lines. These unique cell populations appeared to represent the characteristics of the original tumors derived from patients.

**Conclusions:**

We confirmed the existence of transcriptomic ITH in breast cancer PDOs. Some cellular states were commonly observed in multiple PDOs, whereas others were specific to single PDO lines. The combination of these shared and unique cellular states formed the ITH of each PDO.

**Supplementary Information:**

The online version contains supplementary material available at 10.1186/s13058-023-01617-4.

## Background

There is mounting evidence that cancer cell heterogeneity within tumors plays an essential role in part of cancer resistance and recurrence [1, 2]. In breast cancer, stronger intratumor heterogeneity (ITH) in estrogen receptor (ER) expression, as assessed by immunostaining, is associated with a poorer prognosis [3]. ITH in human epidermal growth factor receptor 2 (HER2) amplification and expression also serves as a predictive indicator of poor therapeutic response [4, 5]. Although these studies focused on single genes essential to breast cancer, they were still suggestive of a relationship between cancer cell diversity and its malignant potential. Therefore, we must elucidate the molecular mechanisms underlying ITH and their functional significance to improve therapeutic strategies. However, partly a consequence of the limited number of suitable experimental models for the analysis of ITH, the overall research landscape of ITH in human cancers is still largely unexplored.

In recent years, three-dimensional organoid culture methods have been actively utilized in cancer research [6, 7] because patient-derived organoids (PDOs) are easier to manipulate and can be analyzed more quickly than patient-derived xenografts, yet still retain some of the tissue architecture and biological characteristics of the patients’ tumor of origin [8, 9]. Therefore, the intratumor heterogeneity of cancer cells may be preserved. Although several reports analyze PDOs from the perspective of intratumor heterogeneity in some cancer types, such as lung and colorectal cancer [10, 11], no reports examine the detailed cellular transcriptomic ITH in breast cancer PDOs.

In the present study, we use single-cell RNA-seq (scRNA-seq) to show that breast cancer PDOs are composed of several cell clusters with distinct cellular states. Furthermore, we define a cluster-specific gene signature (ClustGS) for each cell cluster in PDOs, and successfully capture one aspect of ITH. We found that seven ClustGSs were shared among multiple PDOs and some ClustGSs were specific to single PDOs only. The former represent cellular states related to the cell cycle, EMT, and estrogen response, whereas the latter represent cellular states often not clearly defined. These unique gene signatures specific to a certain PDO may reflect the biological characteristics of that PDO, indicating the assessment of cellular transcriptomic ITH may be helpful for characterizing PDOs.

## Methods

### Establishing organoids from clinical specimens

Surgical specimens were obtained by the core needle biopsy of surgically removed tumors. The specimens were dissociated into single cells using a MACS Tumor Dissociation Kit and a gentle MACS dissociator (Miltenyi Biotec, North Rhine-Westphalia, Germany) in accordance with the manufacturer’s instruction. For pleural effusion specimens, drainage fluid was collected after trocar placement, centrifuged, and purified with Red Blood Cell Lysis Solution lysis buffer (Miltenyl Biotec). The organoids were established from the obtained cells as described previously [8]. Briefly, cell pellets were resuspended in BME gel (R&D Systems, Minnesota, USA) at 4 °C and seeded to form domes in 24-well or 48-well plates (IWAKI, Tokyo, Japan), polymerized at 37 °C for 10–20 min, and the prepared medium was added at a volume of 500 mL per 50 µL BME dome. The medium was changed every 3–4 days and the PDO was passaged depending on growth. Cultrex Organoid Harvesting Solution (Thermo Fisher Scientific, Massachusetts, USA) and 0.25 w/v% Trypsin-1 mM EDTA 4Na Solution with Phenol Red (FUJIFILM, Tokyo, Japan) were used for passaging of organoids. Expression of ER and PgR in surgical specimens was assessed using the Allred score [12].

### Single-cell RNA-seq experiment

Organoids were dissociated into a single-cell suspension using harvesting solution and trypsin, and the cells were cryopreserved for the scRNA-seq experiment. Moreover, BC specimens were dissociated into single cells, as described above. The scRNA-seq libraries were prepared using the BD Rhapsody Single-Cell Analysis system (BD, New Jersey, USA) in accordance with the manufacturer's instruction. Briefly, after thawing, cells were labeled with BD Single-Cell Multiplexing Kit. The labeled cells were washed, pooled, and loaded onto the BD Rhapsody microwell cartridge; then cDNA was synthesized using the BD Rhapsody Whole Transcriptome Analysis Amplification Kit. The resulting gene expression and cellular label libraries were sequenced on Illumina NextSeq 550 platform (Illumina, California, USA) with paired-end reads (read1, 75 bp; index1, 8 bp; read2, 75 bp). Sequencing data were processed using the BD Rhapsody Analysis pipeline on the Seven Bridges Genomics platform and converted to a gene expression count matrix.

### Data analysis

We utilized the web-based analysis pipeline for BD Rhapsody and BD Precise ASSAYS (https://www.sevenbridges.com/bdgenomics/) to generate the count matrixes and Seurat [13] for downstream analysis. RSEC counts were used as the input count matrixes. Low-quality cells with over 40% of mitochondrial RNAs and < 400 or > 9000 features were filtered out. To avoid misassignment between cells and samples, we performed pre-clustering of each PDO using Seurat’s standard flow and manually removed clusters that exhibited distant embedding from major clusters and different distribution of each sample tag that were possible contaminants from other samples.

For high-quality scRNA-seq data of each PDO, transcripts count matrixes were normalized to the total number of counts for the cell and multiplied by a scaling factor of 10,000. The normalized values were subsequently natural-log transformed using Seurat’s “NormalizeData()” function and a linear transformation was applied by “ScaleData()”. The principal component analysis was performed by “RunPCA()” with top 2,000 highest variable features identified by “FindVariableFeatures()” with vst selection method. We excluded PCs that contained ribosomal protein-encoding genes in more than half of the top 20 features. Then we performed Seurat’s standard clustering procedures using “FindNeighbors()” and “FindClusters()” with the top 20 PCs and a resolution of 0.4. To visualize data, “RunUMAP()” was used with the same PCs to identify the clusters.

After clustering of each PDO, we identified differentially expressed features as cluster-specific gene expression signatures (ClustGS) by using the “FindAllMarkers()” function with “only.pos = TRUE, min.pct = 0.25, logfc.threshold = 0”. We removed mitochondrial RNAs or ribosomal protein-encoding genes and selected the genes with an adjusted *P*-value of less than 0.05. The top 25 most significant genes in each cluster of each PDO were selected as ClustGS. Any clusters with fewer than 25 significant genes (adjusted *P*-value < 0.05) were not considered for further analysis. Meta-clusters were manually defined by the Jaccard similarity index. All genes included in at least two ClustGS were defined as meta-cluster specific genes (meta-ClustGS).

Gene enrichment analysis for each ClustGS was performed using the “enrichr()” function of ClusterProfiler v3.18.1 [14] with MsigDB Hallmark genes obtained through msigdbr’s msigdbr(species = “Homo sapiens,” category = “H”) function. The false discovery rate (FDR) was used as the adjusted *P*-value**.**

Gene overlap analysis between ClustGS and cluster-specific expressed genes of corresponding primary tumors or different passage organoids was performed using “GeneOverlap” package’s newGeneOverlap() and testGeneOverlap() functions. To obtain cluster-specific expressed genes in each dataset, we selected the genes with < 0.05 adjusted P-values calculated by Seurat’s FindAllMarkers() with options of “only.pos = TRUE, min.pct = 0.25, logfc.threshold = 0”.

### Cytokine measurement

The extracellular cytokines in the culture medium were comprehensively semi-quantified using the Proteome Profiler Array Human XL Cytokine Array Kit (R&D Systems) in accordance with the manufacturer’s instruction. Briefly, culture medium from four organoids (PDOs 155, 165, 166, 180) was collected 72 h after the change of fresh medium and frozen at − 20 °C. Between 59.5 and 846.2 µL of the culture medium, normalized by RNA quantity from the PDOs in the same culture, was loaded into the cytokine array. The Odyssey XF Imaging System (LI-COR, Nebraska, USA) was used to detect fluorochromes; the absorbance was set to 685 nm and the exposure time was set to 10 min. The signal intensity corresponding to each factor in the array was quantified by ImageJ software, and the expression level was calculated relative to the positive control.

### Immunohistochemistry

Tumor sections were stained with hematoxylin–eosin (HE) and immunohistochemically examined for CHI3L1 and CST3 using a Nichirei Histofine system (Nichirei Biosciences Inc.). Tissue sections were incubated with primary anti-CHI3L1 antibody (Cell Signaling, #47,066) and anti-CST3 antibody (Abcam, ab109508) at a 1:800 and 1:4000 dilution, respectively, and detected with HRP-labeled polymer-conjugated secondary antibody (Histofine Simple Stain MAX PO, multi, Nichirei). Color development was achieved with 3,3′-diaminobenzidine tetrahydrochloride. Organoids were fixed and paraffin-embedded using Epredia HistoGel Specimen Processing Gel (Thermo Scientific) and Tissue-Tec VIP (SAKURA, Japan). Immunohistochemical staining on the organoid sections was performed by incubating with anti-CHI3L1 antibody (Cell Signaling, #47,066) and anti-CST3 antibody (Abcam, ab109508) at a 1:400 and 1:500 dilution, respectively. Signals were detected using Dako EnVision + Dual Link System-HRP (Agilent).

## Results

### Organoids established from breast cancer tissues contained morphologically heterogeneous cell populations

**Fig. 1:**
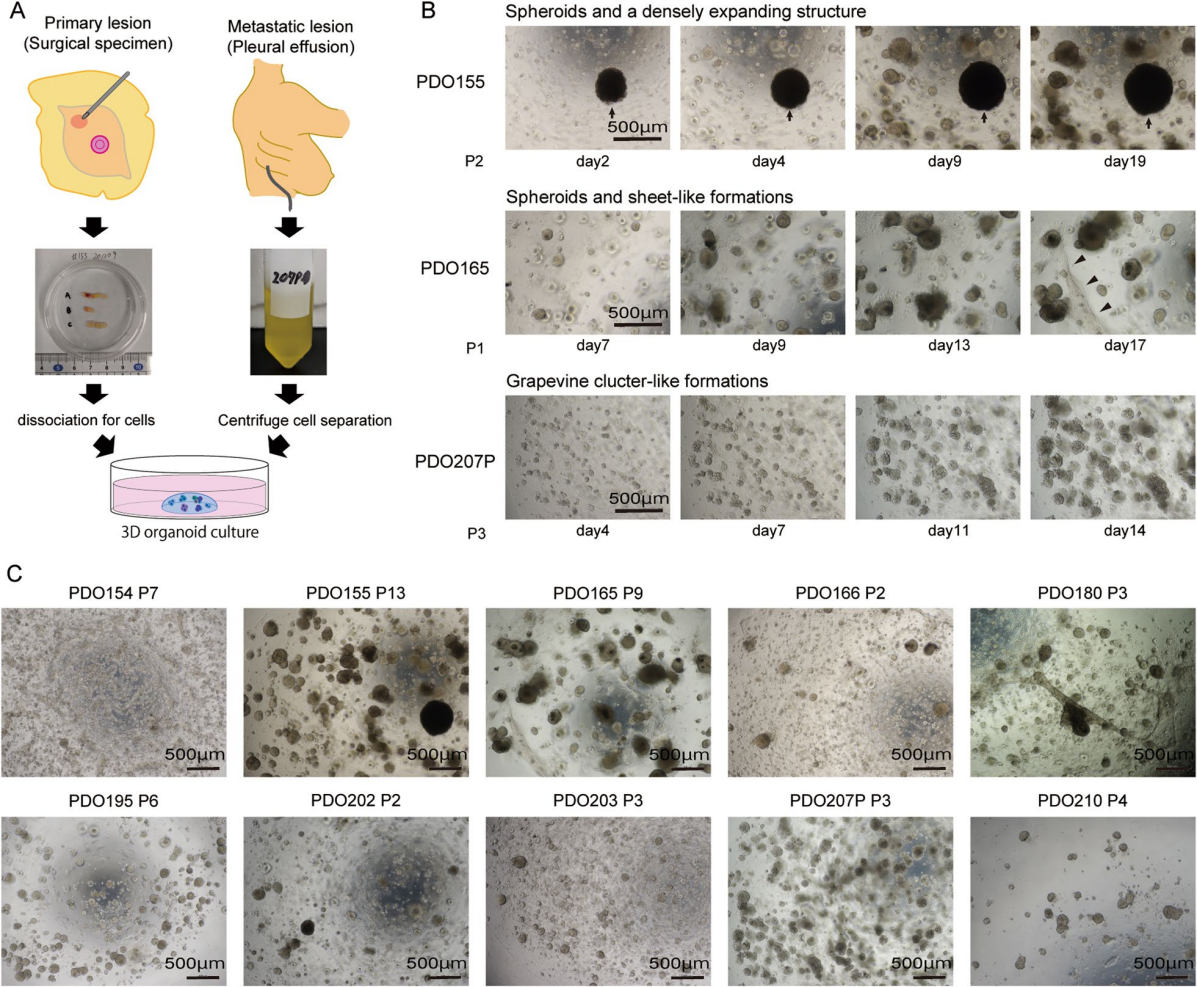
Process for establishing PDOs.** A** Schematics for collecting clinical specimens and establishing organoids. **B** Representative images showing the changes in the morphology of organoids over time. Arrow: densely expanding structures. Arrowhead: sheet-like formations around the spheroids. **C** Morphology of the 10 PDOs. The number after the P represents the number of passages of indicated PDO

Ten PDO lines established during a certain period of time were subjected to the analysis in this study (Fig. 1A). The clinicopathological features of the 10 cases are presented in Table 1. Briefly, eight were from patients with primary breast cancer, one case (PDO210) was local recurrence in the chest wall after total mastectomy, and one case (PDO207P) was lung metastasis with pleural effusion. In addition, one of the eight cases of primary breast cancer (PDO155) developed metastasis soon after surgery. Tumors with high proliferative potential may have been more frequently established as organoids; there were five cases with nuclear grade 3 and Ki67 > 20%.

**Table 1:** Clinicopathological characteristics of 10 patients

PDO	Age	Menopausal	Location	Histological type	ER*	PgR*	HER2	Grade	Ki67	LVI**
154	53	Pre	Breast	IDC***	4 + 2	3 + 2	+	3	55%	+
155	51	Pre	Breast	IDC	0 + 0	1 + 1	+	3	95%	+
165	41	Pre	Breast	IDC > IMPCa***	5 + 3	5 + 3	−	3	35%	+
166	48	Pre	Breast	IDC	0 + 0	0*0	+	3	65%	−
180	32	Pre	Breast	IDC	5 + 3	5 + 3	−	1	25%	+
195	46	Pre	Breast	IDC	5 + 2	5 + 3	−	2	10%	−
202	45	Pre	Breast	IDC	5 + 3	4 + 3	−	2	35%	−
203	53	Post	Breast	IMPCa > IDC	5 + 2	4 + 3	−	1	5%	−
207P	52	Post	Pleural effusion	Adenocarcinoma	N/A^†^	N/A^†^	N/A^†^	N/A^†^	N/A^†^	N/A
210	47	Pre	Chest wall	IDC	4 + 2	4 + 2	−	3	90%	−

*Assessed by Allred Score = Proportion score + Intensity score

**LVI Lymphovascular invasion

***IDC Invasive ductal carcinoma, *IMPCa* Invasive micropapillary carcinoma

†Diagnosed by cytology

The speed of organoid growth differed for each PDO. PDO155, 166, and 207P had the fastest growth rate, with a doubling time of approximately 7 days, which continued even after ten passages. PDO 165 and 180 grew more slowly than the three PDO lines listed above, with a doubling time of approximately 14 days. The other lines grew more slowly.

Organoid morphology was also heterogeneous among PDOs (Fig. 1B, C). Spheroids were common in all PDOs, but some PDOs had mixed populations of organoids with different shapes. For example, PDO155 consisted of two different cell populations: spheroids and densely expanding structures. PDO154, 165, and 180 appeared to form sheet-like structures around the spheroids that dragged each spheroid along, altering their 3D position every few days. PDO207P contained grapevine cluster-like formations. PDO166, 195, 202, 203, and 210 formed homogenous spheroids.

Collectively, our results present the inter- and intratumor heterogeneity for organoid growth and morphology, suggesting that the PDOs reflect the biological properties of the original tumors.

### Examining ITH of PDOs by single-cell transcriptome profiling

We performed single-cell transcriptome analysis of 10 PDOs by multiplexing scRNA-seq using the BD Rhapsody Single-Cell Analysis System. After assignment and quality filtering, we obtained the high-quality expression profiles of 9548 cells: an average of 6635 counts and 2203 genes per cell (Additional file 1: Figure S1; “Method” Section). For simple quality assessment, we used the Seurat package to cluster the cells and examine the expression of marker genes (Additional file 1: Figure S1). Almost all cells expressed epithelial markers such as *EPCAM* and keratins, and we considered that the PDO lines used in this study contained nearly no stromal cells. Therefore, we hereby note that the subsequent analysis assesses the ITH of cancer cells.

**Fig. 2:**
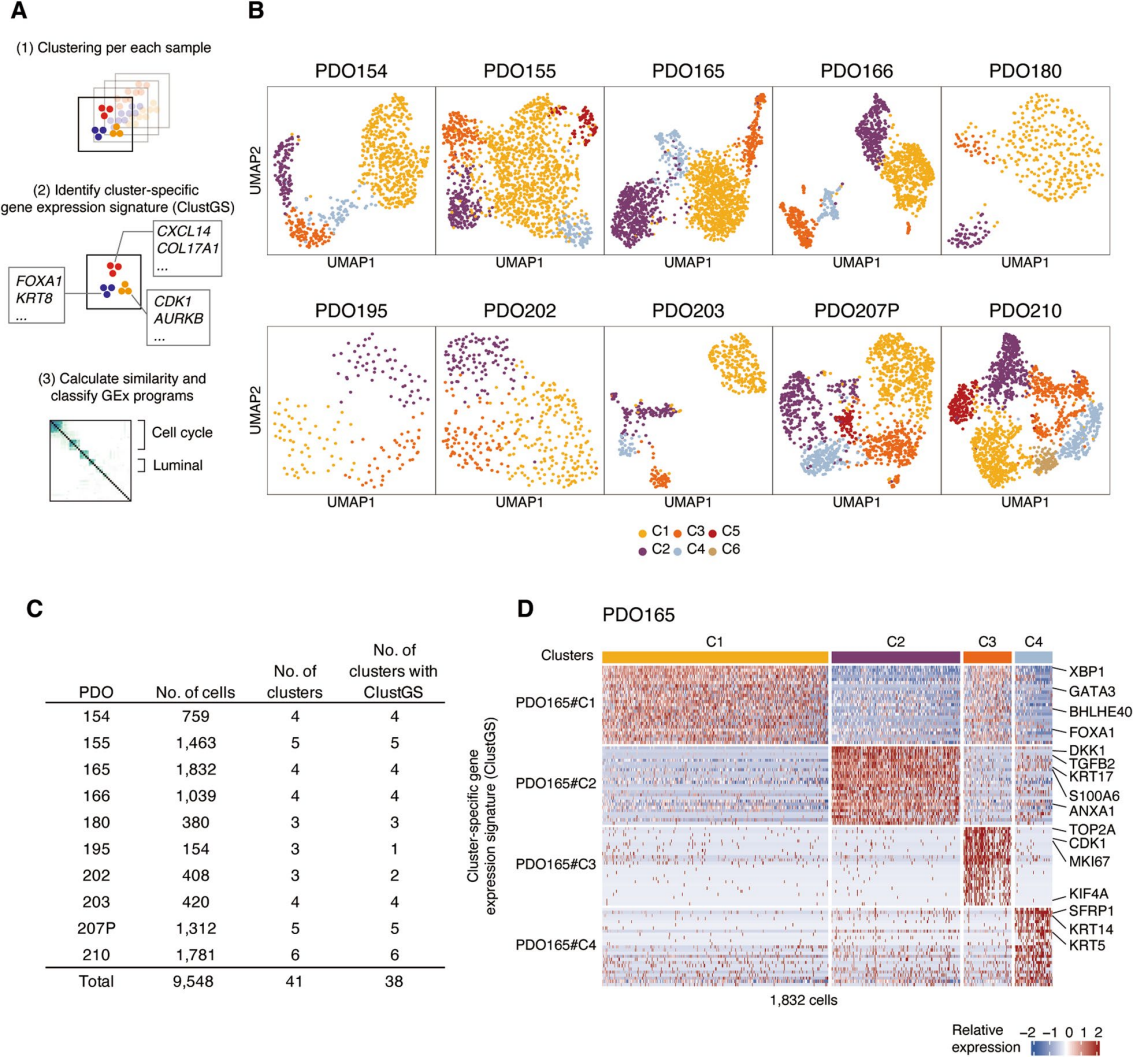
Single-cell transcriptome analysis of 10 PDOs. **A** Schema of analytical flow. **B** Uniform manifold approximation and projection (UMAP) after clustering of scRNA-seq profiles from 10 PDOs. **C** Summary of the number of cells and clusters of each PDO. **D** Heatmap of scaled expression of cluster-specific gene expression signatures (ClustGSs)

The cancer cells were clustered as per patient samples in scRNA-seq analysis (Additional file 1: Figure S1), which is consistent with previous reports [15–18]. To investigate the ITH in each PDO, we developed the analytical approach as follows: (1) cluster the cells in each PDO, (2) identify cluster-specific expressed genes, and (3) calculate their similarity and classify clusters (Fig. 2A). Through a standard analytical flow in Seurat, the cells were classified into 3–6 clusters per each PDO (3 clusters in PDO180, 195, and 202; 6 clusters in PDO210) (Fig. 2B, C). Thus, we identified a set of genes expressed specifically in each cluster within each PDO and defined the most significant 25 differentially expressed genes as the cluster-specific gene signature (ClustGS). Any clusters with fewer than 25 significant genes (adjusted *P*-value < 0.05) were not considered for further analysis. Each PDO had at least one cluster with ClustGS, and in total, 38 clusters with a ClustGS were identified (Fig. 2C).

For example, PDO165 had four clusters with a ClustGS (Fig. 2D). ClustGSs in PDO165#C1 contained luminal-lineage transcription factors (TFs) (*FOXA1*, *GATA3*); PDO165#C2 contained *TGFB2* and *DKK1*; PDO165#C3 contained cell cycle-related genes (*CDK1*, *MKI67*); and PDO165#C4 contained basal keratins (*KRT5*, *KRT14*) (Fig. 2D; Additional file 2: Table S1). Consistently, the molecular signatures database (MSigDB) hallmark gene enrichment analysis suggested that PDO165#C1 was related to estrogen response; PDO165#C2 and PDO165#C4 were related to the epithelial–mesenchymal transition (EMT); and PDO165#C3 was related to the cell cycle (Additional file 1: Figure S2). These results suggest that each PDO is composed of multiple subpopulations with distinct cell states and lineages with the cell cycle, estrogen response, and EMT-like gene expression programs.

We next assessed whether the ITH observed in the PDOs reflected the ITH in the original tumor tissues and whether it was maintained after repeated PDO passages. We performed scRNA-seq experiments on two additional original breast cancer tissues (165 and 210) and two different passages of PDO lines (180 and 210). Subsequently, we performed the same analysis as above, and confirmed the existence of ITH in each sample (Additional file 1: Figure S3; Additional file 2: Table S2). Comparison of the characteristics of the identified cell populations by looking at the overlap of cluster-specific expressed genes revealed that they were composed of somewhat similar cell populations between original tissues and PDOs as well as between different passages of the same PDO lines (Additional file 1: Figure S4; Additional file 2: Table S3), although not completely one-to-one correspondence. Thus, we decided to analyze these PDOs further, considering that they retain some extent of ITH of the original tissues even after successive passages.

### Identifying cell populations defined by common expression programs across PDOs

**Fig. 3:**
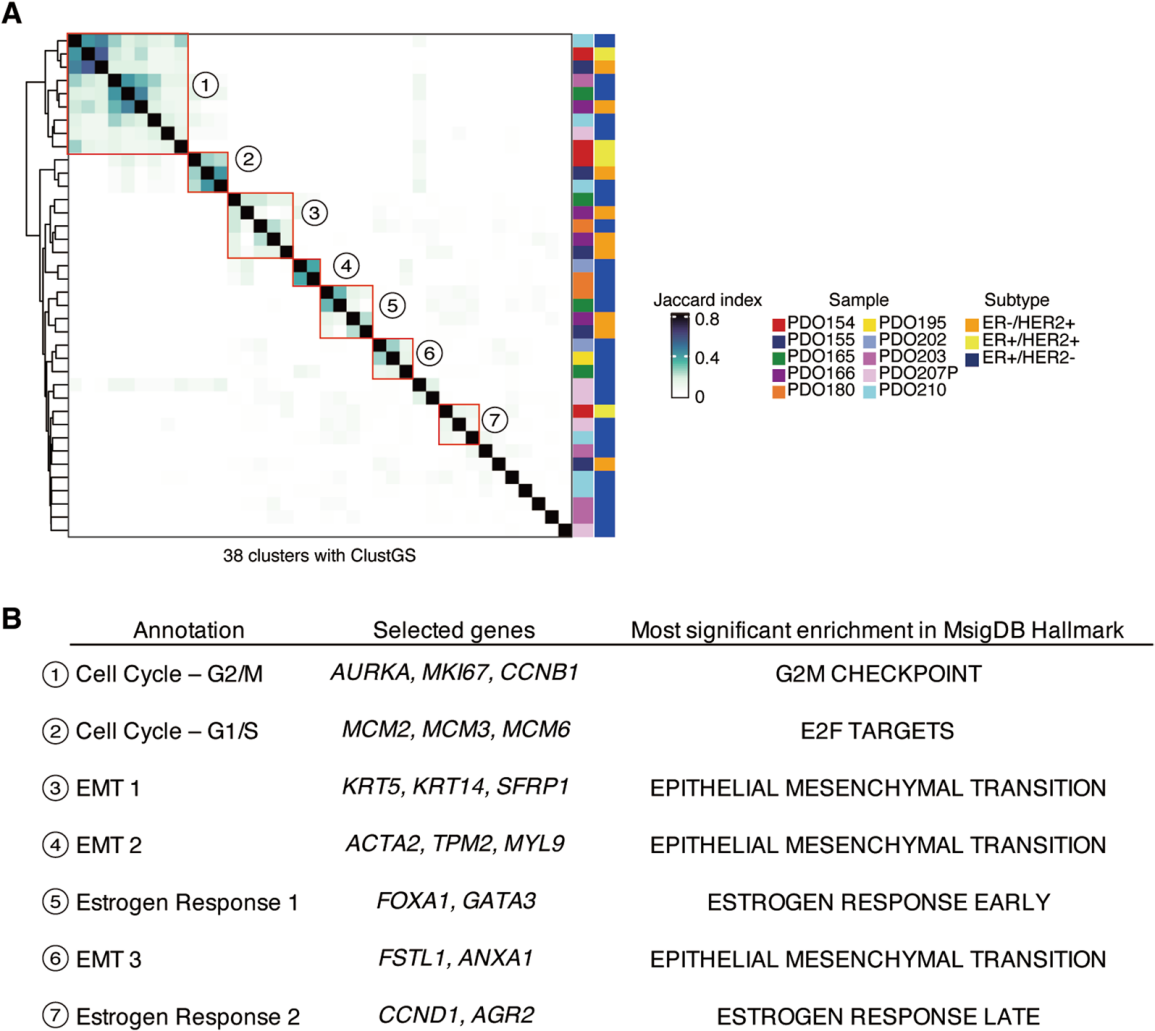
Meta-clusters designated by common expression signatures.** A** Heatmap of the Jaccard similarity index for 38 ClustGSs. Right annotations represent PDO samples and receptor status of the original tumors. **B** Annotation for meta-clusters and profiling of meta-ClustGSs

To compare the degree of ITH between PDOs, we calculated the Jaccard similarity index across 38 ClustGS from each cluster. Based on the similarity of ClustGS, seven meta-clusters were identified with the hierarchical clustering guide, representing a set of cell clusters characterized by the same expression programs across PDOs (Fig. 3A). To annotate each of the meta-clusters, we selected the overlapping genes across ClustGS in at least two PDOs as a meta-cluster (“meta-ClustGS”; Additional file 2: Table S4). Gene enrichment analysis for meta-ClustGSs using the MsigDB Hallmark gene set suggested that meta-ClustGSs 1–2 were associated with cell cycle, notably the G2/M phase for meta-ClustGS 1 and G1/S phase for meta-ClustGS 2; meta-ClustGSs 3, 4, and 6 were associated with the EMT; and meta-ClustGSs 5 and 7 were associated with estrogen response (Fig. 3B; Additional file 1: Figure S5). Although meta-ClustGSs 3, 4, and 6 all contained EMT-associated genes, each gene signature was different: basal cytokeratin (such as *KRT5*, *KRT14*) in meta-ClustGS 3, mesenchymal marker *ACTA2* in meta-ClustGS 4, and the metastasis-associated gene *ANXA1* in meta-ClustGS 6. There were also two meta-ClustGSs associated with estrogen response: luminal-lineage specific TFs *FOXA1* and *GATA3* in meta-ClustGS 5, *CCND1* in meta-ClustGS 7. These results suggested that each PDO contained several cell populations that were regulated by the defined gene expression programs that were common across PDOs.

### Common gene expression programs highlighted ITH across PDOs

**Fig. 4:**
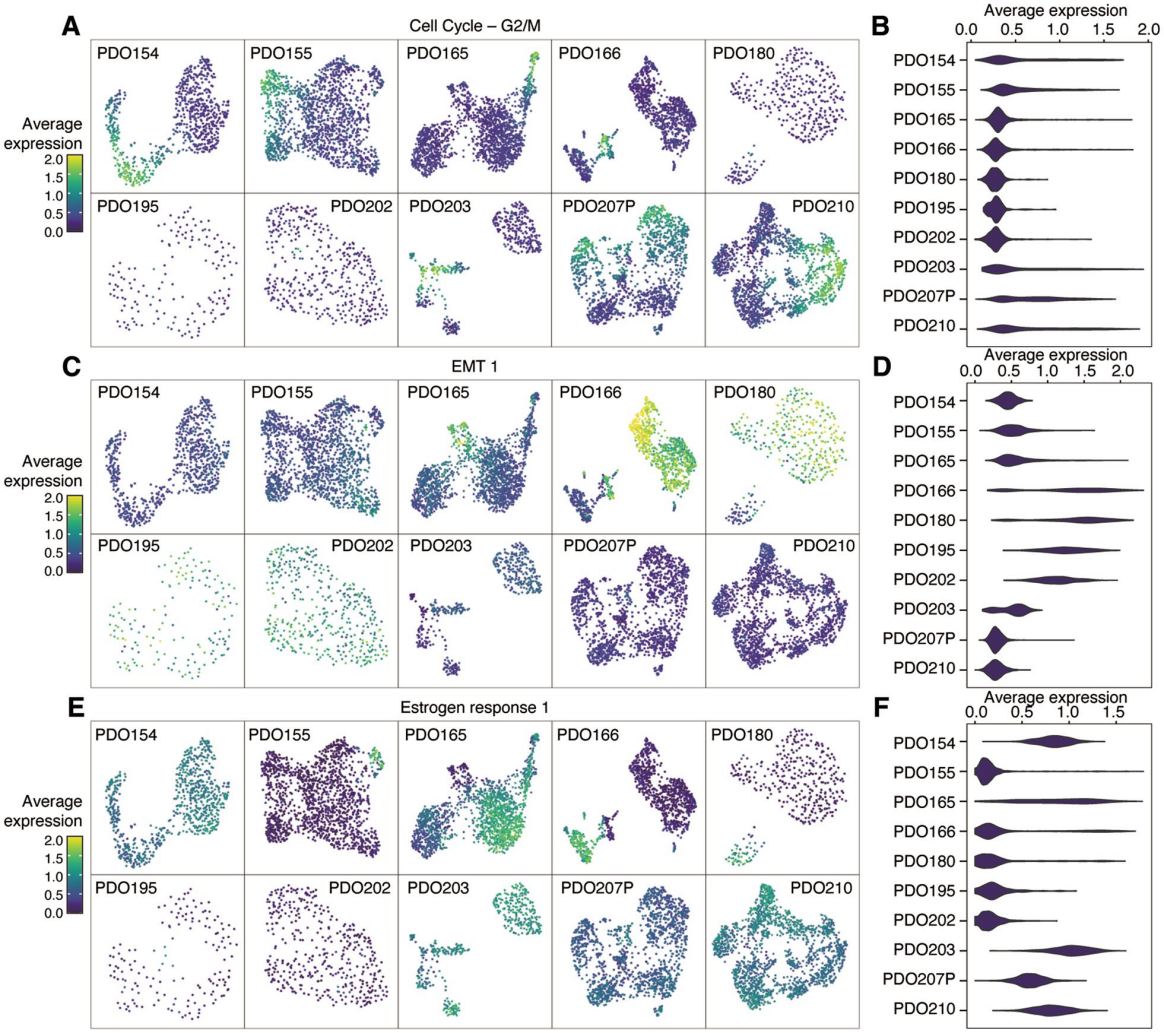
Expression status of meta-ClustGSs in each PDO. **A**, **C**, **E** UMAP overlay representing the expression levels of meta-ClustGS 1 (cell cycle, G2/M phase) in (**A**), meta-ClustGS 3 (EMT1) in C, and meta-ClustGS 5 (estrogen response 1) in (**E**). **B**, **D**, **F** Violin plot representing expression levels of meta-ClustGS 1 (cell cycle, G2/M phase) in B, meta-ClustGS 3 (EMT1) in D, and meta-ClustGS 5 (estrogen response 1) in (**F**)

We subsequently characterized each PDO using meta-ClustGSs. The meta-ClustGS 1 (Cell cycle—G2/M) was present in 7 of 10 PDO lines (Figs. 3A, 4A, B; Additional file 1: Figure S6), indicating that the proliferating cell populations were common in PDOs. Interestingly, both meta-ClustGS 3 (EMT1) containing basal markers (*KRT5* and *KRT14*) and meta-ClustGS 5 (estrogen response 1) containing luminal-lineage TFs (*FOXA1* and *GATA3*) were observed in four PDO lines (PDO155, 165, 166, and 180) derived from patients with ER+ /HER2– or ER–/HER2+ tumors, suggesting that these PDOs had distinct ITH of co-existing of basal-like and luminal cells (Fig. 4C–F; Additional file 1: Figure S6). Clusters of cells with meta-ClustGS 5 expression were observed in PDO155 derived from ER–/HER2+ tumors, suggesting that a small number of cells expressed ER target genes that were not detected by the bulk assessment, i.e., immunohistochemistry, but were observable at a single-cell resolution (Fig. 4E; Additional file 1: Figure S6). Most cells in three PDOs (PDO180, 195, and 202) derived from ER+ tumors expressed the meta-ClustGS 3 (EMT1, basal-like), implying that the property of ER+ cancer cells was altered in organoid culture or that part of organoids was derived from juxta-tumoral basal epithelial cells in these PDOs (Fig. 4C, D).

Another estrogen response-related meta-cluster, meta-ClustGS 7, was observed in PDO154, 207P, and 210. Interestingly, they had distinct clinical features such as ER + /HER2 + (PDO154), pleural effusion sample (PDO207P), and early recurrence (PDO210). These suggested that meta-ClustGS 7 may reflect a different cellular state to normal ER-positive breast cancer cells with a good prognosis, even though it is annotated with the same estrogen response by MsigDB analysis. For example, meta-ClustGS 7 contained *CCND1*, which has been implicated in endocrine resistance and poor outcome [19, 20], suggesting that this gene signature might reflect the peculiarity of a cell population (Additional file 1: Figure S6).

These results indicate that meta-ClustGSs can describe each PDO with a qualitative and quantitative assessment of ITH.

### PDO-specific cell clusters reflected distinct properties for PDOs

**Fig. 5:**
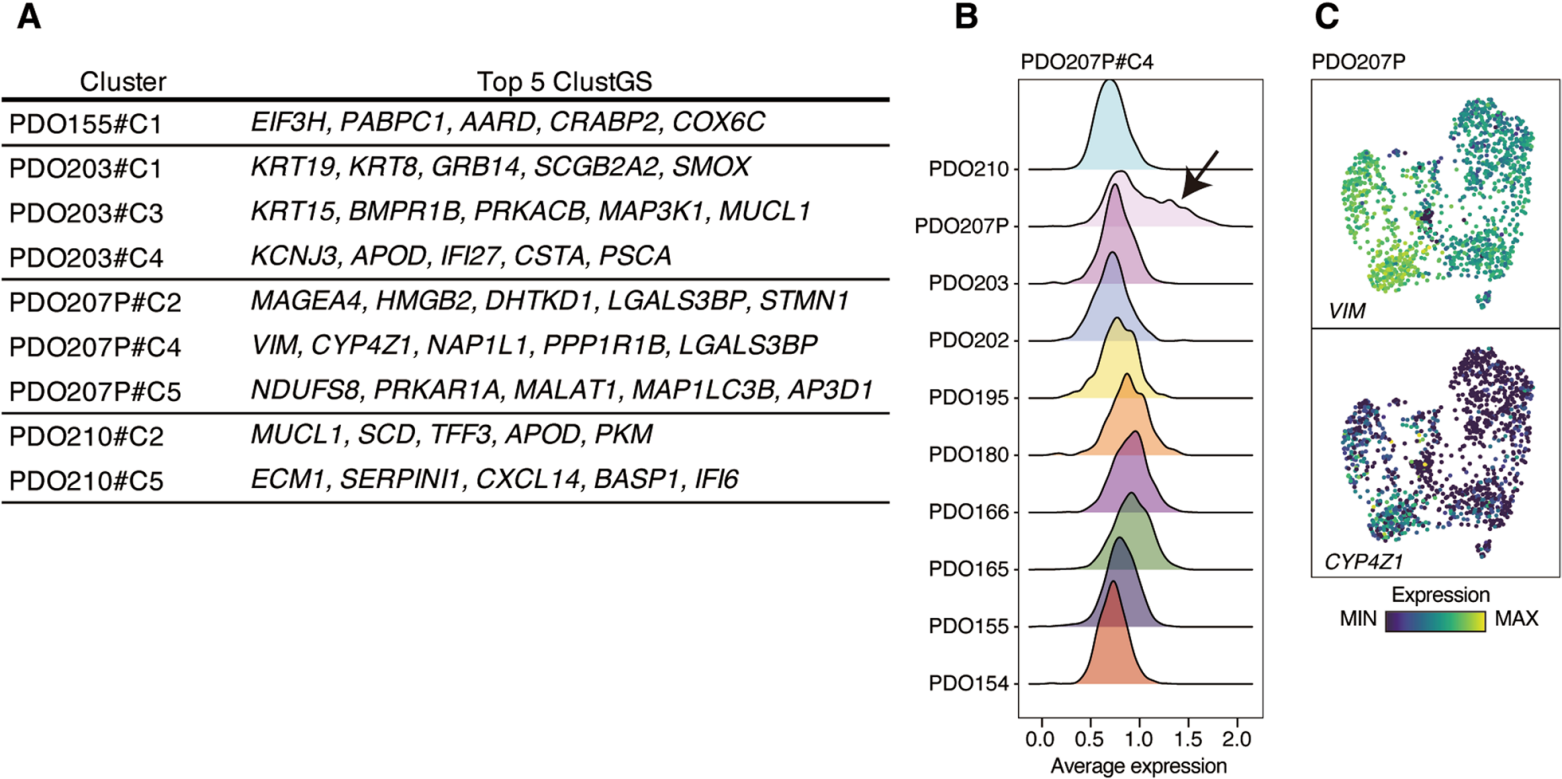
PDO-specific ClustGSs. **A** Summary for nine PDO-specific ClustGSs. **B** Ridge plot of expression level of PDO207P#C4 ClustGS. Black arrows show a cell population with high expression of the ClustGS. **C** UMAP overlay representing expression levels of PDO207P#C4 ClustGS

In the process of identifying meta-clusters, we also identified nine PDO-specific clusters (Fig. 5A; Additional file 2: Table S5). These ClustGSs corresponded to four PDO lines, each derived from cases with characteristic clinical courses or histopathology: PDO155 from inflammatory breast cancer (IBC), PDO203 from invasive micropapillary carcinoma, PDO207P from metastatic breast cancer, and PDO210 from recurrent breast cancer. Thus, ClustGSs not shared with any other PDOs possibly reflected the clinical or molecular features for each PDO.

For example, PDO207P#C4 ClustGS included the mesenchymal marker *VIM* and *CYP4Z1*, which are associated with poor prognosis and high-grade tumors in breast cancer [21]. This ClustGS was highly expressed in a certain population of PDO207P (Fig. 5B, C). As PDO207P was derived from the metastatic pleural effusion, this cluster probably reflected the distinct features of mesenchymal and aggressive disease in PDO207P.

### Organoids from a patient with inflammatory breast cancer

We further investigated PDO155 which was derived from a patient with IBC. The patient developed liver metastases early after surgery and had a poor clinical outcome [22, 23]. To our knowledge, no reports are available on PDO lines being established from patients with IBC and then precisely analyzed. We therefore considered that it might be possible to find the biological features of IBC in the PDO155. We focused on a specific cell population, PDO155P#C1 (Fig. 2A), with a ClustGS that was not similar to other ClustGSs. In the gene signature, genes associated with RNA transport and translation initiation functions were highly enriched (*EIF3H, PABPC1, EIF3E*, and *EIF2S3*; Additional file 2: Table S5). In this cell population, translation was expected to occur preferentially to cell cycle progression (Fig. 4C), suggesting that cell size may increase [24, 25]. This may be related to the morphological heterogeneity observed specifically in PDO155, in which some of the organoids formed gigantic spheroids (Fig. 1B, C).

**Fig. 6:**
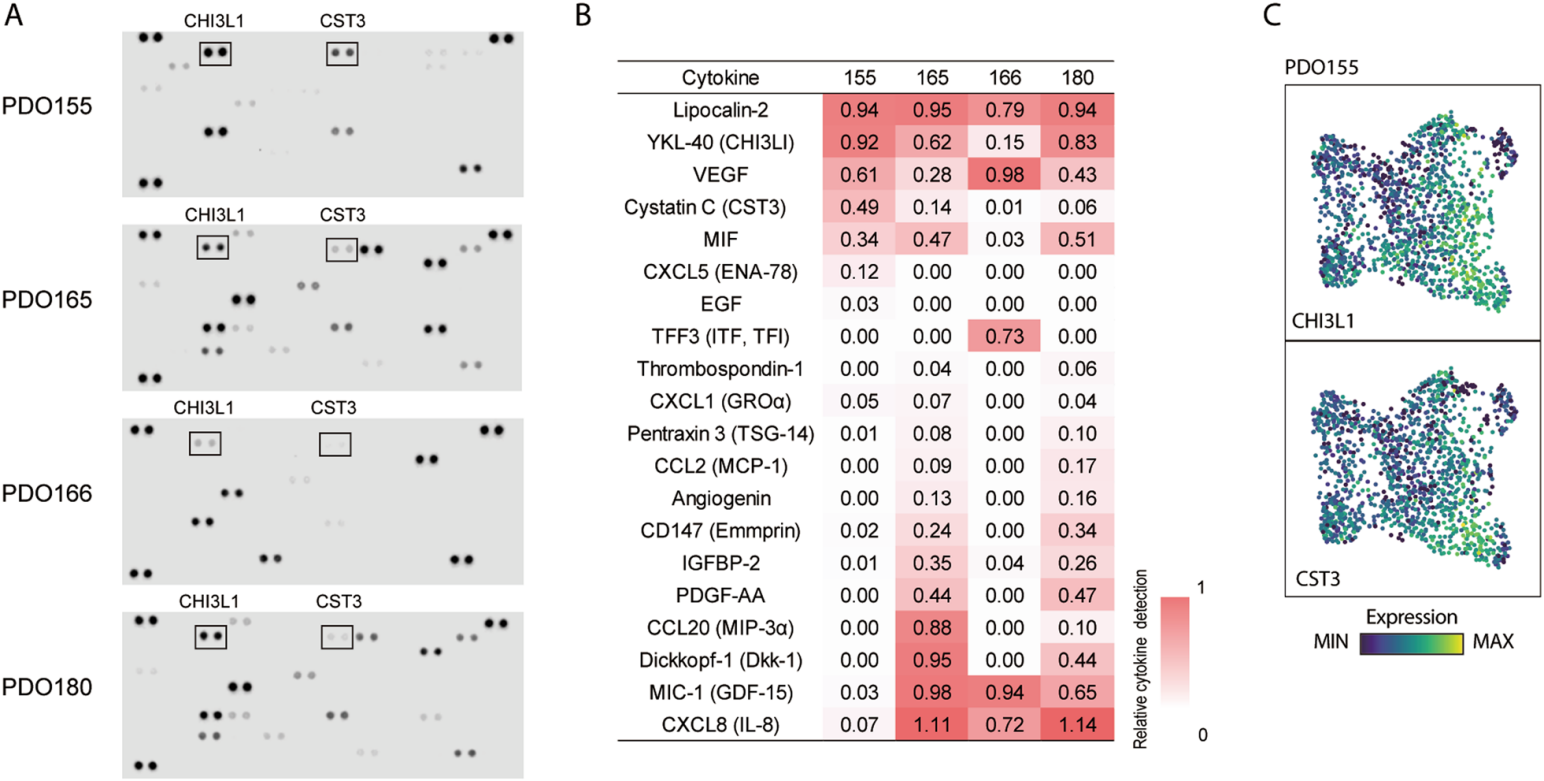
Measurement of cytokines in PDO culture medium. **A** Cytokine arrays measured 102 cytokines in culture supernatant from the indicated PDOs. Positive controls at the upper left, upper right, and lower left edges and negative controls at the lower right edge. **B** Heatmap of the quantitative results of detected cytokines relative to the positive control. **C** UMAP overlay representing expression levels of the indicated genes

The gene signature also included several genes encoding cytokines, such as *CST3* and *CHI3L1*. We thought that the specific cytokines released from cancer cells might be related to the pathophysiological characteristics of IBC. Thus, we examined cytokines in the culture medium in PDO155 and other PDOs using a cytokine array (Fig. 6A). *CST3* and *CHI3L1* were highly detected in PDO155; in particular, the secretion of CST3 was specifically observed in PDO155. CST3 is known to have various functions on cancer progression [26, 27], which may be related to the feature that part of PDO155 forms huge clumps. Interestingly, these genes were not uniformly expressed in all cells; they were heterogeneously and non-randomly expressed (Fig. 6B). By performing immunohistochemistry of PDO155 and pathology specimens of this patient, we confirmed that CST3 and CHI3L1 proteins were heterogeneously expressed in PDO155 and even in the original tumor tissue (Additional file 1: Figure S7). This indicates that only a subset of the cell population may only exhibit the specific characteristics observed in the bulk assay; conversely, a unique feature visible in the ITH may display the representative feature of that PDO.

## Discussion

This study aimed to investigate the intratumor transcriptomic heterogeneity in breast cancer PDOs. We performed scRNA-seq analysis and found that PDOs were composed of 3–6 cell populations with distinct cellular states. Furthermore, we characterized the cellular states of these cell populations based on the top 25 genes expressed specifically in each population, which we defined as the cluster-specific gene signature (ClustGS). We then compared the cellular states within and between PDOs by examining the similarity of the ClustGSs. Seven meta-ClustGSs, reflecting cellular states, were common among multiple PDOs, and nine ClustGSs were found to be unique to individual organoids. The common meta-ClustGSs included cell states related to the cell cycle, EMT, and estrogen response. In contrast, the cellular states unique to the individual PDOs appeared to display unique characteristics for that specific PDOs. These results indicated that we may be able to estimate the unique characteristics of a particular PDO by examining the ITH within the PDO; a comparison with other PDOs may not be necessary.

Using single-cell RNA-seq technology, we confirmed the existence of ITH in breast cancer PDOs. Several studies have reported the ITH of cancer cells and its characteristics in cancer tissues and cell lines [15, 16, 18, 28]. Kinker et al. [28] reported 12 recurrent heterogeneous programs, including cell cycle, EMT, stress, and senescence. They also demonstrated that many transcriptomic ITH patterns of cancer cells reflected the intrinsic plasticity of cancer cells, even outside of the native microenvironment. Our data show ITH related to cell cycle and EMT within breast cancer PDOs, which is consistent with the above study. In addition, several PDOs also displayed ITH related to estrogen response and basal cells, which may be specific to the lineage plasticity of breast cancer.

We also observed patient-specific ITH patterns in several PDOs. Previous studies tended to focus on the recurrent patterns of ITH and ignore unique ITH [28, 29]. However, we hypothesized that the patient-specific ITH might represent the unique characteristics of the PDO and contain useful information. As the ITH pattern in cancer cells is thought to reflect the cell-of-origin and the activity of the lineage specific transcription factors [2], the ITH pattern should provide some information on the origin or pathophysiology of the cancer cell population.

From this perspective, we focused on PDO155 derived from a patient with aggressive inflammatory breast cancer (IBC). IBC is a cancer subtype with a poor prognosis characterized by extensive lymphatic embolism and cutaneous inflammation, and its etiology and molecular mechanisms are poorly understood [23, 30]. PDO155 was distinct from the other nine organoids in terms of morphology. It grew quickly, and some of the organoids became huge (Fig. 1B, C). These unique characteristics of morphology may possibly reflect the nature of IBCs, which tend to form emboli in the lymphatic vessels. The scRNA-seq analysis also identified cell populations in a particular state that were not observed in other organoids, which may hold some clues. The signature (PDO155P#C1 ClustGS) included several genes related to translation. PDO155 had *MYC* amplification (data not shown) and may have enhanced ribosome biogenesis [31]. The abnormal increase in translation may be related to the increase in cell size and the giant organoids [24, 32]. Although further functional verification is needed, these data indicate that the specific ITH pattern may predict and explain the characteristics of the original IBC.

## Conclusions

We described the landscape of transcriptomic ITH within breast cancer PDOs. We observed cell populations with cellular states common to various PDOs and cell states specific to individual PDOs. The latter could reflect the unique biology of the tumor of origin.

## Supplementary Information

**Additional file 1**.** Figure S1**: A and B. QC metrics showing unique feature counts and percentage of mitochondrial RNA per cell. C. UMAP visualization of scRNA-seq profiles from 10 PDOs. Each dot represents a single-cell colored by its corresponding PDOs. D and E. UMAP overlay representing expression levels of *EPCAM* and *KRT8*. ** Figure S2**: Hallmark terms and adjusted p-values detected by MSigDB hallmark gene enrichment analysis for each ClustGS in PDO165. ** Figure S1**: A and C. UMAP visualization of 1,822 and 6,362 cells annotated as EPCAM-positive cells within scRNA-seq profiles of primary breast cancer tissues of patients 165 (A) and 210 (C). B and D. Heatmaps showing the number (top) and significance (bottom) of overlapping genes between each ClustGS identified in indicated PDOs and each cluster-specific expressed gene list identified in its original tumor tissues. ** Figure S4**: A and B. UMAP visualization of scRNA-seq profiles of PDO180 P6 (A) and PDO210 P8 (B). C and D. Heatmaps showing the number (top) and significance (bottom) of overlapping genes between each ClustGS identified in indicated PDOs and each cluster-specific expressed gene list identified in PDOs. ** Figure S5**: Hallmark terms and adjusted p-values detected by MSigDB hallmark gene enrichment analysis for 7 meta-ClustGSs. ** Figure S6**: Heatmap showing which meta-ClustGS are present or absent in each PDO. ** Figure S7**: HE staining and immunohistochemistry (CHI3L1 and CST3) of PDO155 and its original tumor tissue.

**Additional file 2**.**Table S1**: 38 ClustGSs across 10 PDOs.**Table S2**: Cluster-specific expressed gene lists in P165 and P210.**Table S3**: Cluster-specific expressed gene lists in PDO180 P6 and PDO210 P8.**Table S4**: Meta-ClustGSs.**Table S5** PDO-specific ClustGSs.

## Data Availability

R code for performing the analyses in available at (https://github.com/KoheiKumegawa/BC_10PDOs_scRNA). Processed scRNA-seq data are available from the lead contact upon request.

## Abbreviations

ACTA2: Actin alpha 2, smooth muscle
ANXA1: Annexin A1
CCND1: Cyclin D1
CDK1: Cyclin-dependent kinase 1
CHI3L1: Chitinase 3 like 1
ClustGS: Cluster-specific gene signature
CST3: Cystatin C
CYP4Z1: Cytochrome P450 family 4 subfamily Z member 1
EIF2S3: Eukaryotic translation initiation factor 2 subunit gamma
EIF3E: Eukaryotic translation initiation factor 3 subunit E
EIF3H: Eukaryotic translation initiation factor 3 subunit H
EMT: Epithelial–mesenchymal transition
EPCAM: Epithelial cell adhesion molecule
ER: Estrogen receptor
FOXD1: Forkhead box protein A1
GATA3: GATA binding protein 3
HER2: Human epidermal growth factor receptor 2
IBC: Inflammatory breast cancer
ITH: Intratumor heterogeneity
KRT5: Keratin 5
KRT14: Keratin 14
MKI67: Marker of proliferation ki-67
MSigDB: Molecular signatures database
MYC: MYC proto-oncogene, bHLH transcription factor
PABPC1: PolyA binding protein cytoplasmic 1
PDO: Patient-derived organoid
PgR: Progesterone receptor
scRNA-seq: Single-cell RNA-seq
TF: Transcription factors
VIM: Vimentin
