# Supplementary material for: Transcriptomic intratumor heterogeneity of breast cancer patient-derived organoids may reflect the unique biological features of the tumor of origin
Source: Breast Cancer Res. 2023 Feb 21;25:21. doi: 10.1186/s13058-023-01617-4 (PMC9942352; doi:10.1186/s13058-023-01617-4)
Supplement: Supplementary file 1 — Additional file 1. Figure S1: A and B. QC metrics showing unique feature counts and percentage of mitochondrial RNA per cell. C. UMAP visualization of scRNA-seq profiles from 10 PDOs. Each dot represents a single-cell colored by its corresponding PDOs. D and E. UMAP overlay representing expression levels of EPCAM and KRT8. Figure S2: Hallmark terms and adjusted p-values detected by MSigDB hallmark gene enrichment analysis for each ClustGS in PDO165. Figure S1: A and C. UMAP visualization of 1,822 and 6,362 cells annotated as EPCAM-positive cells within scRNA-seq profiles of primary breast cancer tissues of patients 165 (A) and 210 (C). B and D. Heatmaps showing the number (top) and significance (bottom) of overlapping genes between each ClustGS identified in indicated PDOs and each cluster-specific expressed gene list identified in its original tumor tissues. Figure S4: A and B. UMAP visualization of scRNA-seq profiles of PDO180 P6 (A) and PDO210 P8 (B). C and D. Heatmaps showing the number (top) and significance (bottom) of overlapping genes between each ClustGS identified in indicated PDOs and each cluster-specific expressed gene list identified in PDOs. Figure S5: Hallmark terms and adjusted p-values detected by MSigDB hallmark gene enrichment analysis for 7 meta-ClustGSs. Figure S6: Heatmap showing which meta-ClustGS are present or absent in each PDO. Figure S7: HE staining and immunohistochemistry (CHI3L1 and CST3) of PDO155 and its original tumor tissue. [file 13058_2023_1617_MOESM1_ESM.pdf]

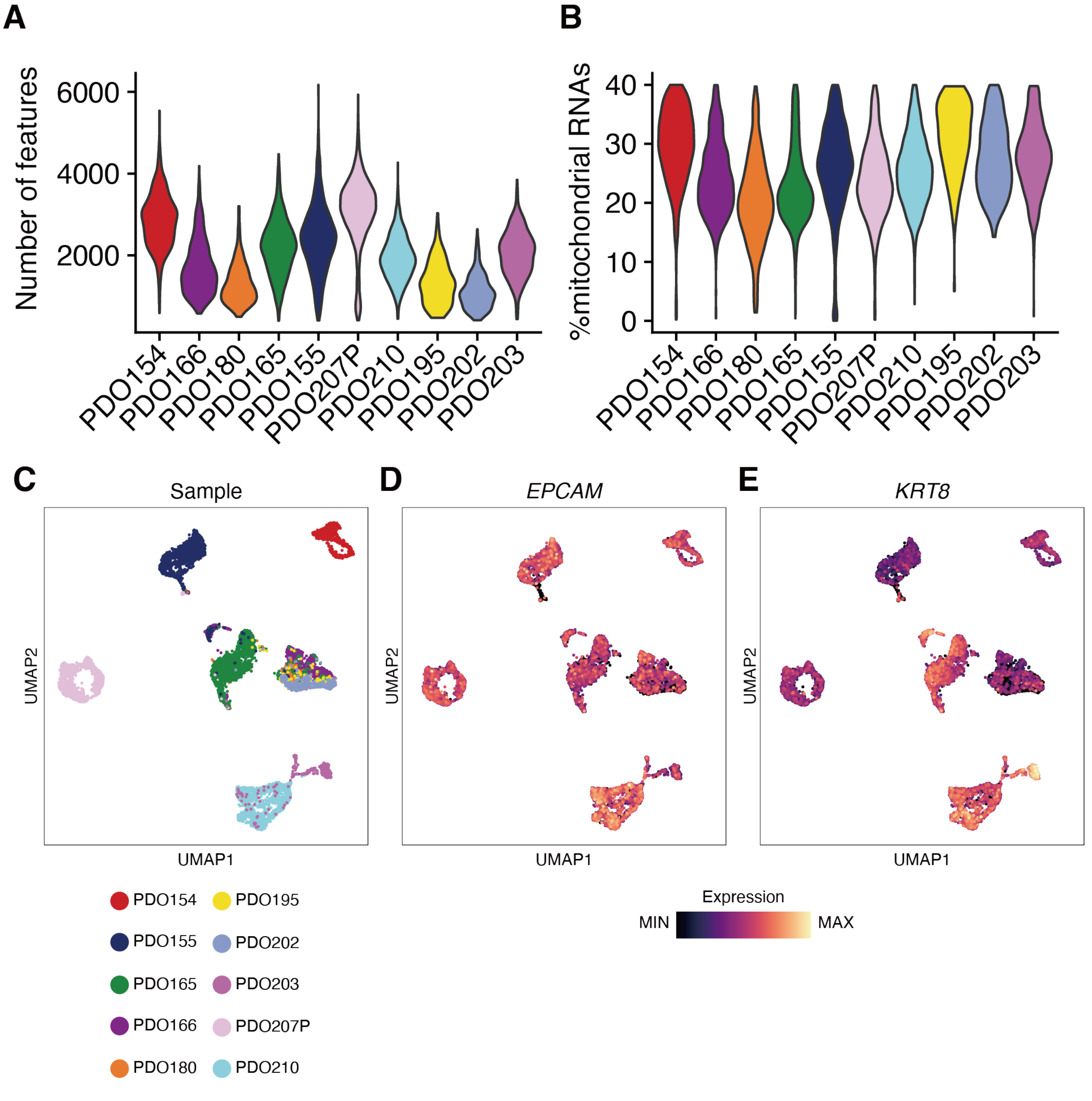

Supplementary Figure 1

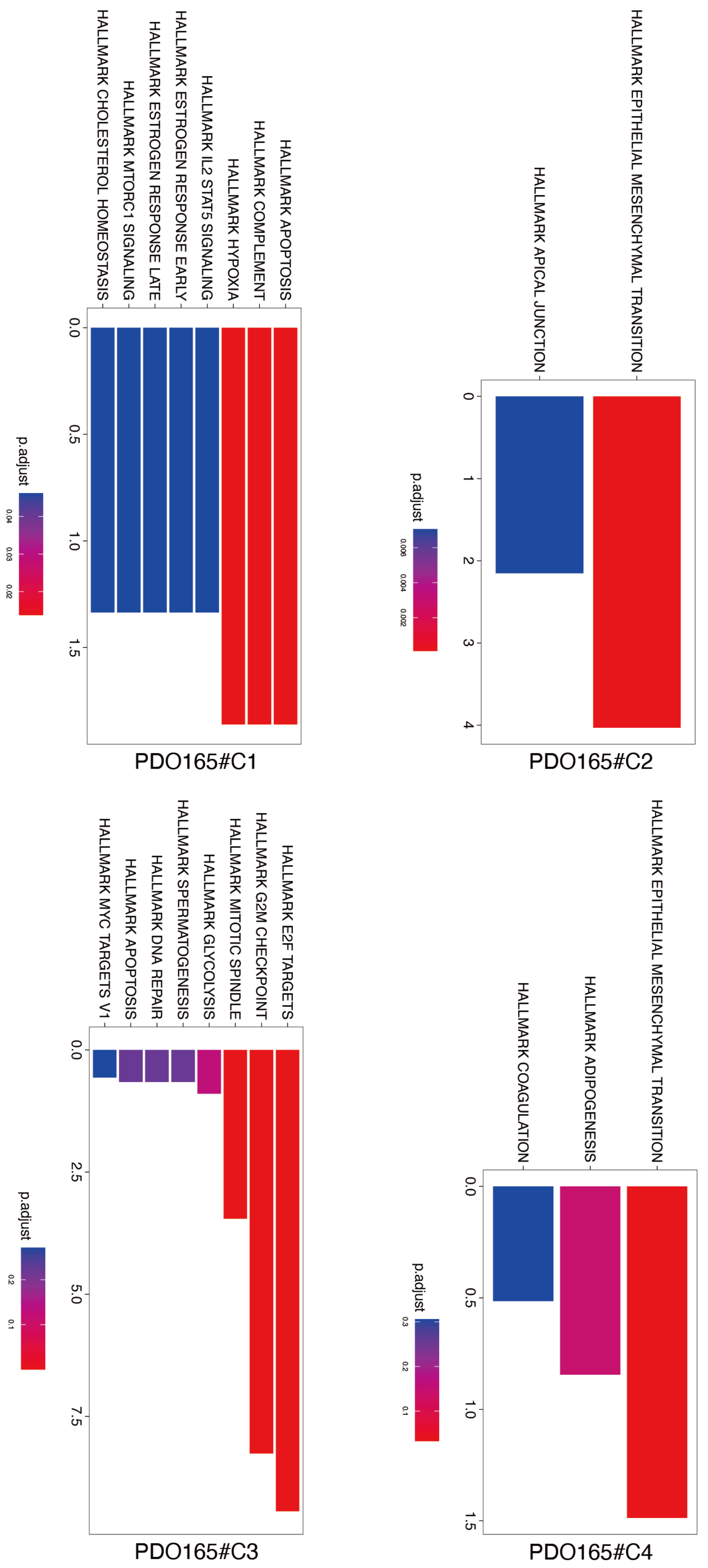

Supplementary Figure 2

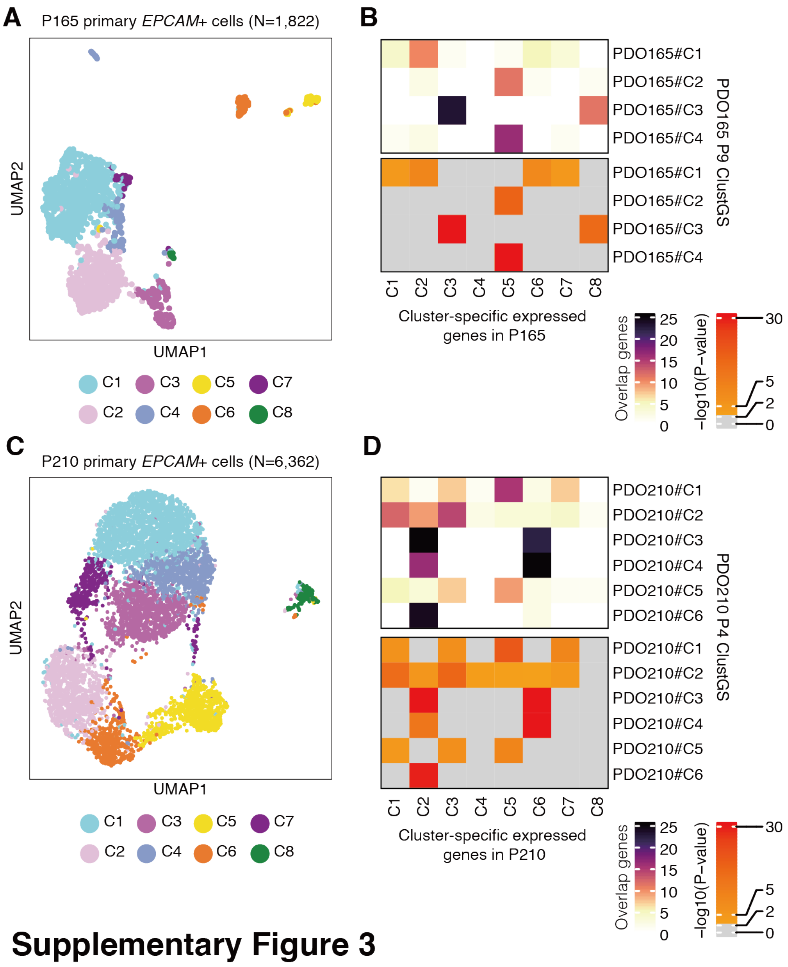

Supplementary Figure 3

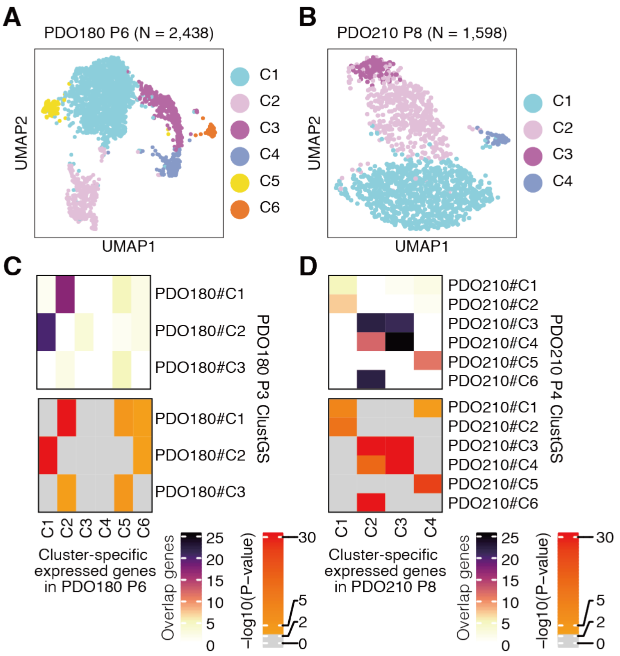

Supplementary Figure 4

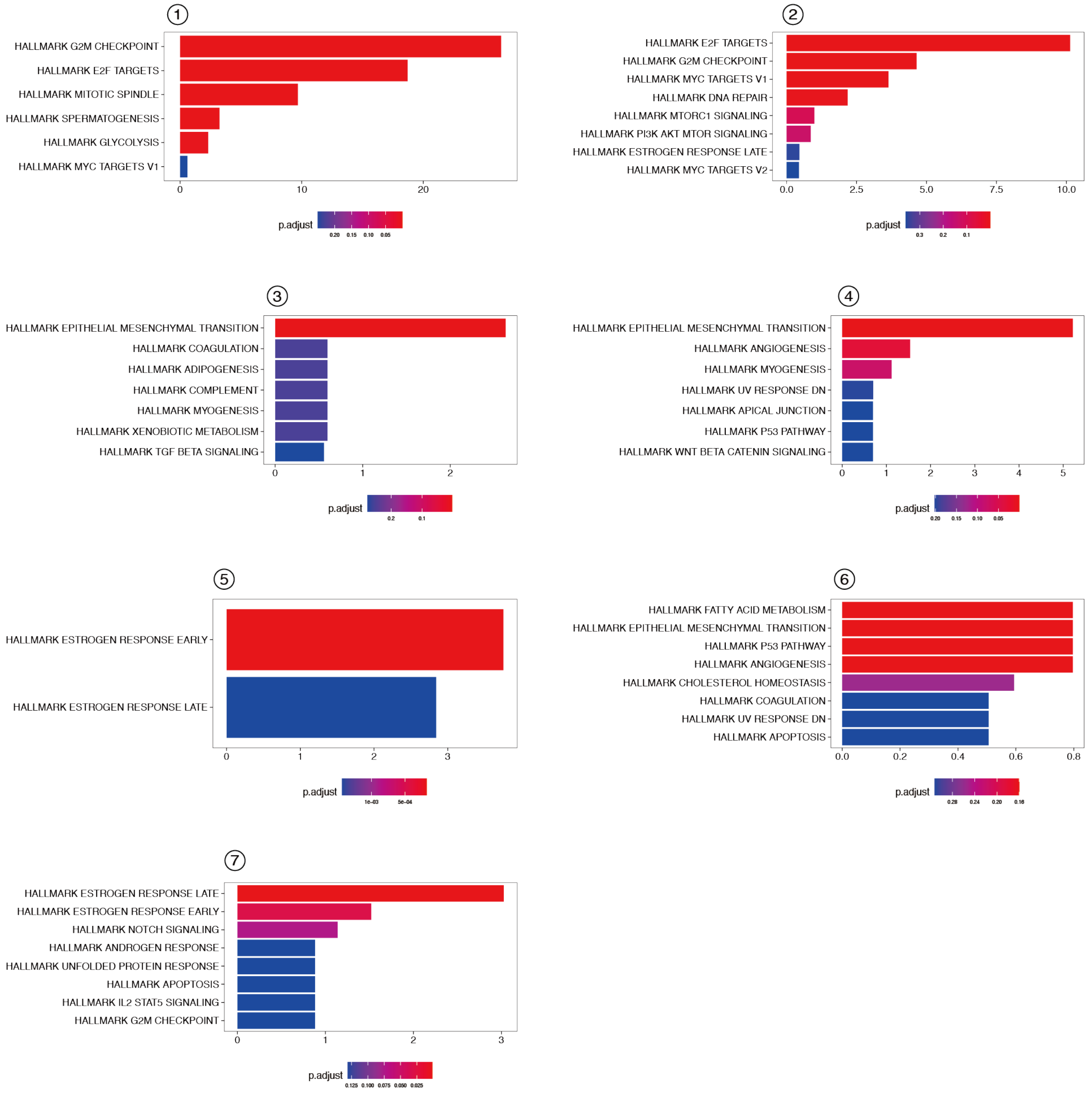

Supplementary Figure 5

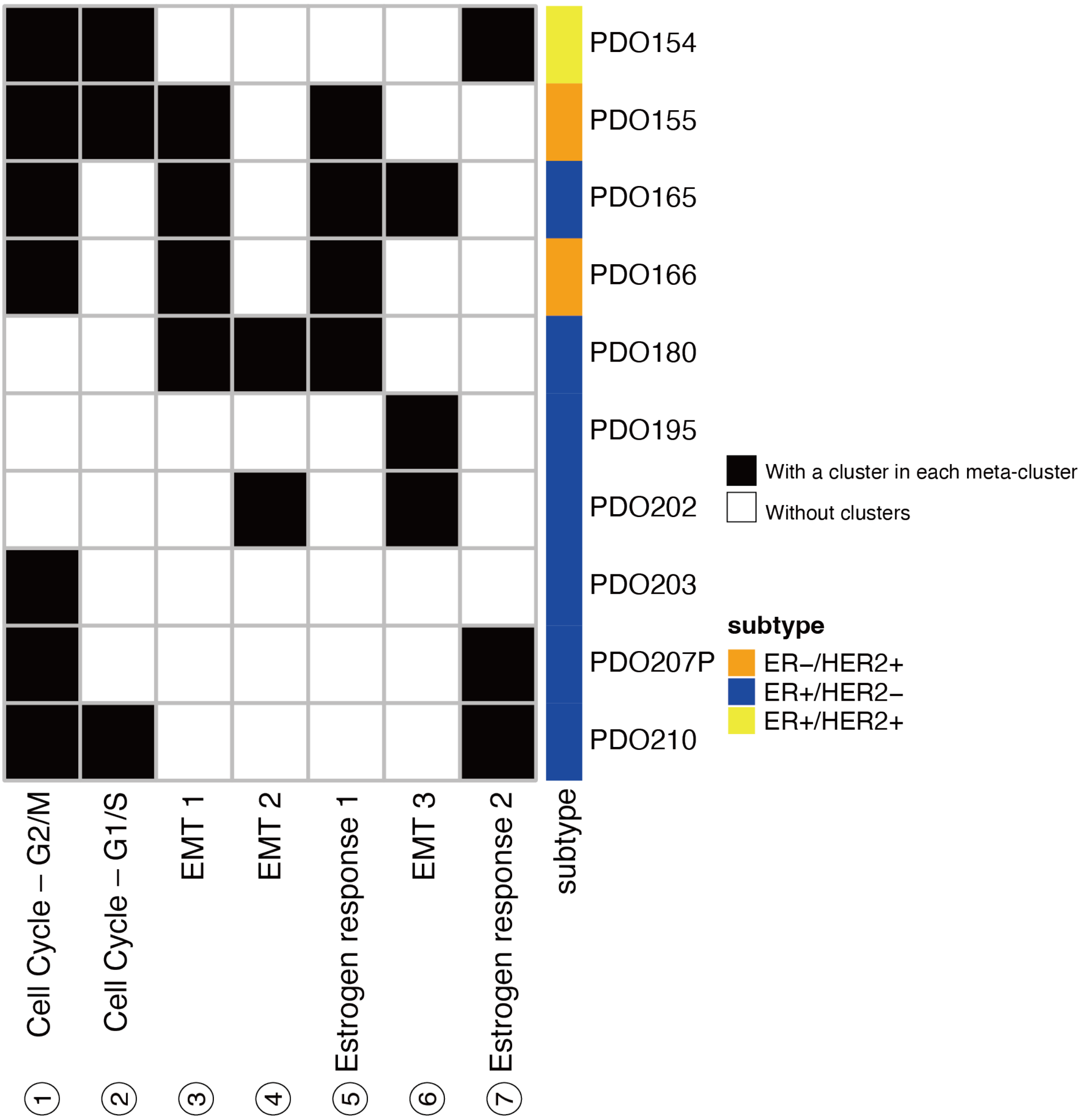

Supplementary Figure 6

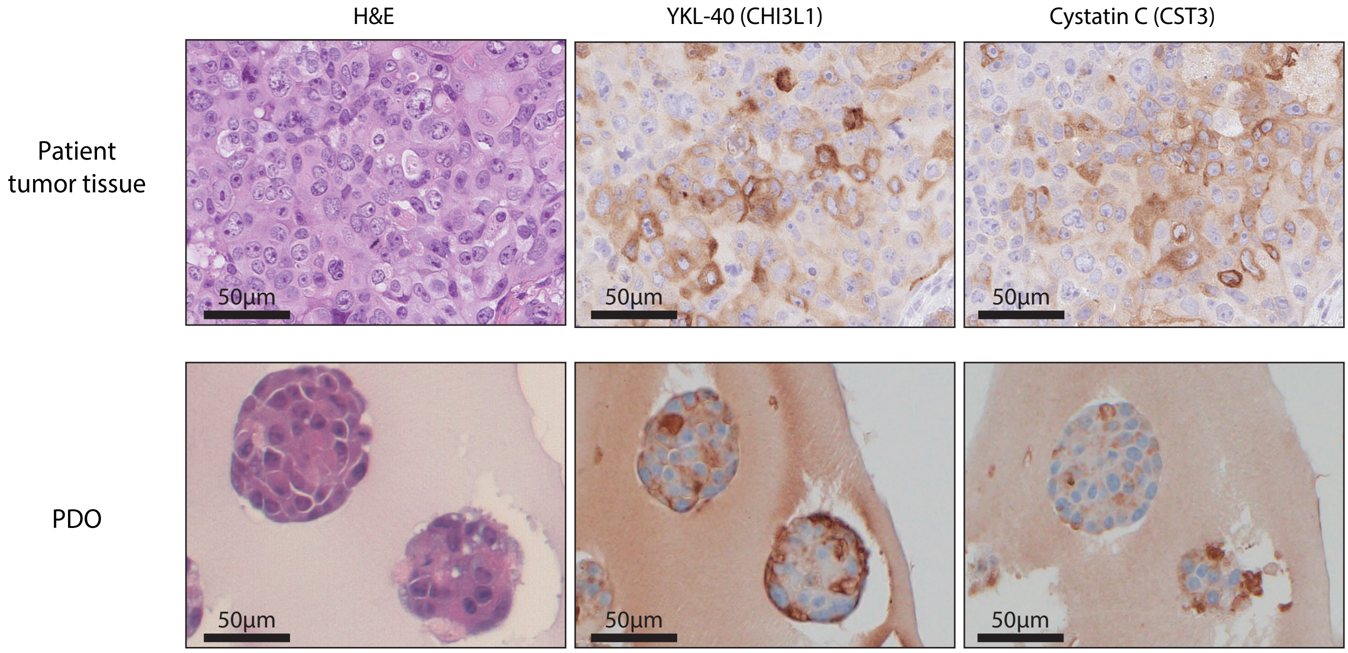

Supplementary Figure 7
